# Supplementary material for: Learning curve and surgical outcome of robotic assisted colorectal surgery with ERAS program
Source: Sci Rep. 2022 Nov 29;12:20566. doi: 10.1038/s41598-022-24665-w (PMC9709162; doi:10.1038/s41598-022-24665-w)
Supplement: Supplementary file 1 — Supplementary Information 1. [file 41598_2022_24665_MOESM1_ESM.zip › LSC Raw data-Ñ[▒K/049/OP_ñw╝╨░O▒Kñσ.pdf]

正式摘要(電子簽章：000000177522927)

W52 / W52

臺中榮民總醫院

Operation Note

[redacted]

- 手術日期：2019/10/08
- 手術醫師：VS 林俊余，CR 黃俋霖，CR 楊曙榮，R 許菀寧
- Anesthesia：GE
- Position：Supine
- Aseptic agents：Soap，Betadine Aqua

Diagnosis

- Pre-operative diagnosis : Cecum tumor.
- Post-operative diagnosis : Ditto.

Surgical Indication

- Remove A-colon tumor

Operation

- Laparoscopic right colectomy and anastomosis

Finding

- 1. A 7x6cm Borrmann type II ulcerative tumor at ascending colon, which invaded through muscular layer, but didn't penetrate through serosa. No enlarged lymph nodes at mesocolon. cT3N0M0.
  2. No other gross tumor or polyp was found.
  3. There was no visible liver metastasis or visible peritoneal seeding.
  4. Some adhesion between A-colon and abdomen wall

Procedure

- General endotracheal anesthesia was induced and then the patient was placed in the supine position. Preoperative antibiotics were given. A Foley catheter and nasogastric tube were placed. The abdomen was prepped and draped in standard sterile fashion.

  A small vertical incision was made alone the umbilicus and a 12mm trocar was inserted into the peritoneal cavity by open method. Pneumoperitoneum was established with CO2 inflation. Laparoscope was inserted and then a 12mm trocar were inserted over LUQ of abdomen. Two 5mm trocars were inserted over RUQ and LLQ of abdomen. The liver, omentum and peritoneum were inspected for evidence of metastatic disease. No metastatic disease was noted. The small bowel was inspected and retracted to downward by grasper. The peritoneum overlying the mesentery was then scored with electrocautery and the ileocolic artery was identified, ligated with endoclips, and transected. The remaining vessels to the cecum were also divided and ligated. Using electrocautery, omentum was freed from the transverse colon and lesser sac was entered. The colon was freed from its peritoneal attachments along the line of Toldt from the cecum to the hepatic flexure. Hemostasis was checked in the operative field. Cecum, ascending colon and hepatic flexure were mobilized and then laparoscope was removed. One midline incision was made at peri-umbilical region. The ascending colon was delivered into the incision and points of transection were selected proximally and distally. Side to side anastomosis was performed with endo GIA 60/3.85. Interrupted 3-0 silk Lambert sutures were used to re-inforcement at outer layer. The anastomosis was checked and found to be intact and widely patent.
  One 12mm penrose drain was placed at sub-hepatic region. The fascia was closed with interrupted sutures of No.1 Vicryl. The fascia of the 12 mm trochar wound was closed with 1-0 Dexon. Subcutaneous tissues were closed with interrupted 3-0 Nylon. The patient tolerated the procedure

- well and was brought to the POR in stable condition.
- Estimated blood loss: minimum
- 器械數量: 正確
- 紗布數量: 正確

Image

- PAINT
  - abdomen

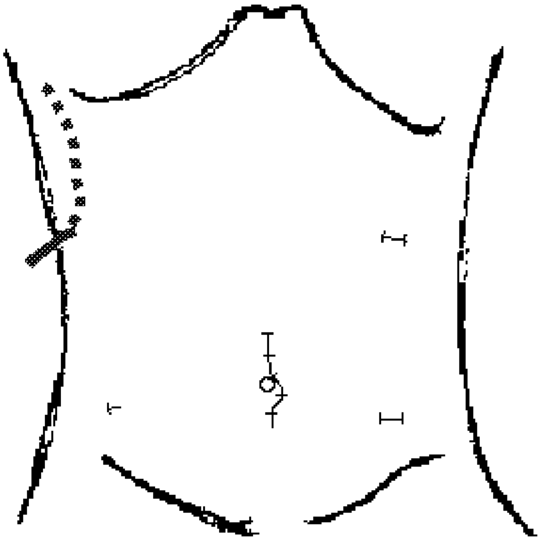

  - colon & rectum

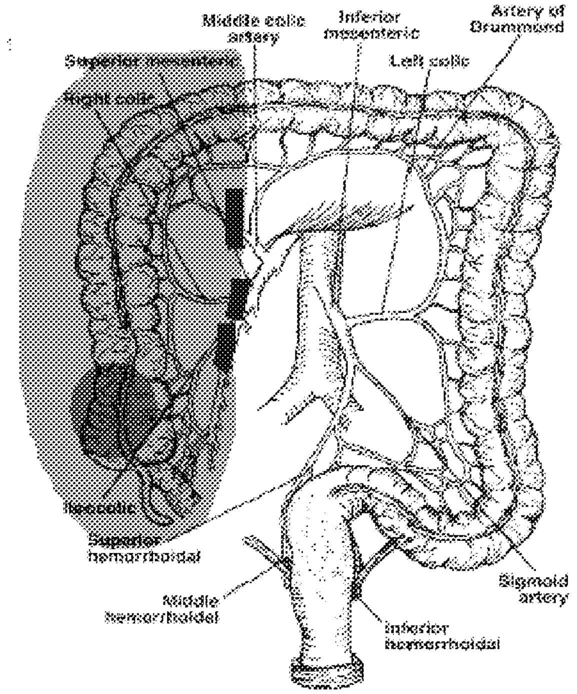

編輯主治 2222H林俊余、主治 2286H黃俋霖

正式摘要(電子簽章：**000000177522927)**

W52 / W52

doctorRank:B
